# Supplementary material for: The genome sequence of the Mallow Seed Moth, Platyedra subcinerea (Haworth, 1828) (Lepidoptera: Gelechiidae)
Source: Wellcome Open Res. 2026 Jul 4;11:413. [Version 1] doi: 10.12688/wellcomeopenres.26937.1 (PMC13562968; doi:10.12688/wellcomeopenres.26937.1)
Supplement: Peer review history

# Reviewer report: Reviewer response for version 1

**DOI:** 10.21956/wellcomeopenres.29656.r166574

**Published:** 2026-09-08

**Authors:** Sarah Inwood

**Affiliations:** 1 University of Otago, Dunedin, New Zealand

This data note describes a reference genome for the Mallow Seed Moth *Platyedra subcinerea*. The haplotype-resolved assembly was generated from PacBio HiFi and Illumina Hi-C reads, and meets all quality benchmarks, with haplotype 1 at chromosome-level. The authors mention that while the genome has not yet been annotated, it will be in the future using RNA-seq data to do so. Overall this is a high-quality assembly, and the report follows the Darwin Tree of Life template.

 Minor comments:

- I agree with the reviewer report from Annabel Whibley that the following sentence feels out of place “It is very local and possibly declining, with a forewing length of 8–9 mm.”
- The software fasta_windows and sanger-tol/curationpretext are included in the software table but are not mentioned in text - if they were used, what for?
- Why are there two to five versions associated with the software BUSCO, samtools and MultiQC? If all versions were used at various stages, it is not made clear to the reader which versions were used for which sections of analysis which reduces the reproducibility of this work

Are sufficient details of methods and materials provided to allow replication by others?

Partly

Is the rationale for creating the dataset(s) clearly described?

Yes

Are the datasets clearly presented in a useable and accessible format?

Yes

Are the protocols appropriate and is the work technically sound?

Yes

Reviewer Expertise:

Genomics, host-parasite interactions, parasitoid wasps, insects, viruses, biocontrol

I confirm that I have read this submission and believe that I have an appropriate level of expertise to confirm that it is of an acceptable scientific standard.

# Reviewer report: Reviewer response for version 1

**DOI:** 10.21956/wellcomeopenres.29656.r162341

**Published:** 2026-07-30

**Authors:** Annabel Whibley

**Affiliations:** 1 Grapevine Improvement, Bragato Research Institute, Lincoln, New Zealand

Hammond and colleagues report the reference genome assembly of the Mallow Seed Moth, *Platyedra subcinerea. * The haplotype-resolved assembly is of excellent quality- as assessed by a suite of evaluation tools. Haplotype 1 has been curated to chromosome level and includes the Z chromosome (the specimen sequenced was male and species ID was verified by barcoding). The report follows standard DToL processes and reporting templates. Metadata is comprehensive, genome evaulations are well-supported and the protocols are appropriated and well-described. I have just the most minor of criticisms: This sentence, which mixes biogeography and morphology, seems an odd juxtaposition *: “It is very local and possibly declining, with a forewing length of 8–9 mm.”*

Are sufficient details of methods and materials provided to allow replication by others?

Yes

Is the rationale for creating the dataset(s) clearly described?

Yes

Are the datasets clearly presented in a useable and accessible format?

Yes

Are the protocols appropriate and is the work technically sound?

Yes

Reviewer Expertise:

Genomics, Bioinformatics

I confirm that I have read this submission and believe that I have an appropriate level of expertise to confirm that it is of an acceptable scientific standard.
